# A Refined Bead-Free Method to Identify Astrocytic Exosomes in Primary Glial Cultures and Blood Plasma

**DOI:** 10.3389/fnins.2017.00335

**Published:** 2017-06-15

**Authors:** Cory M. Willis, Antoine Ménoret, Evan R. Jellison, Alexandra M. Nicaise, Anthony T. Vella, Stephen J. Crocker

**Affiliations:** ^1^Departments of Neuroscience, University of Connecticut School of MedicineFarmington, CT, United States; ^2^Departments of Immunology, University of Connecticut School of MedicineFarmington, CT, United States

**Keywords:** exosomes, astrocytes, multiple sclerosis, flow cytometry, GFAP

## Abstract

Astrocytes are the most abundant glial cell type in the central nervous system (CNS) and are known to fulfill critical homeostatic functions. Dysfunction of activated astrocytes is also known to participate in the development of several neurological diseases. Astrocytes can be uniquely identified by expression of the intermediate filament protein glial acidic fibrillary protein (GFAP). Herein, we report on the development of a rigorous and sensitive methodology to identify GFAP+ exosomes in primary culture using flow cytometry. We then demonstrate that activated astrocytes release increased amounts of exosomes in response to treatment with interleukin-1β. Using this methodology, we report the identification of GFAP+ exosomes in blood and then use a mouse model of inflammatory demyelination, experimental autoimmune encephalomyelitis (EAE), to examine whether the abundance of GFAP+ exosomes in blood circulation changes during clinical illness. We find a detectable increase in the presence of GFAP+ exosomes in EAE mice when compared with non-EAE, control mice. Our data provide a novel perspective on the presence of GFAP in blood as it identifies exosomes as potential astrocyte-derived signals within blood. These data are complementary to previous clinical studies that reported elevated GFAP protein in blood samples from multiple sclerosis (MS) patients during a clinical relapse. These data also reveal the existence of a potential systemic role for astrocyte-derived exosomes in CNS conditions involving inflammation such as multiple sclerosis.

## Introduction

Exosomes are small, extracellular, membrane-bound vesicles (50–150 nm in diameter) secreted by cells as a means of selective transfer of biologically active materials from one cell to another that function as a mechanism of intercellular communication (Guescini et al., 2010; Mause and Weber, 2010; Ostrowski et al., 2010; Gyorgy et al., 2011). The impact of exosomes on physiological functions is mediated, at least in part, by the molecular cargo (e.g., proteins and RNA) within the exosomes themselves. Recent findings demonstrating transport and transfer of proteins and miRNA via exosomes has prompted increased attention on exosomes as potential effectors in diseases as well as the potential to assay exosomes as novel biomarkers for the diagnosis, prognosis and treatment of disease.

Exosomes develop from multivesicular bodies (MVBs), also known as endosomes. These MVBs have a single outer limiting membrane (LM) that surrounds multiple luminal vesicles (LVs). LVs are formed from the LM by inward budding. This process also enables the incorporation of selected membrane proteins onto the surface of the MVBs (Bobrie et al., 2011). Intracellular MVBs have one of two fates: they can either be fully degraded by the lysosomes, or they are shuttled to the plasma membrane where they fuse and release LVs. It is the release of LVs into the extracellular space when these vesicles are called “exosomes” (Colombo et al., 2014). Formation and shedding of exosomes can be regulated by intracellular calcium levels (Wiedmer et al., 1990; Pasquet et al., 1996) or released constitutively, but release can also be stimulated by inflammatory stimuli.

Within the central nervous system (CNS), exosomes are released by all cell types, including neurons, microglia, oligodendrocytes, astrocytes, and neural stem cells (Potolicchio et al., 2005; Kramer-Albers et al., 2007; Basso et al., 2013; Danesh et al., 2014). The contents of exosomes differ depending on cell of origin and pathological setting. For instance, oligodendrocyte exosomes contain myelin proteins unique to oligodendrocytes (Kramer-Albers et al., 2007) and microglial exosomes contain proteins common to immune cells (Potolicchio et al., 2005). Uptake of exosomes by immune cells is a novel and potentially important mode of antigen transfer for MHC presentation that can promote T cell activation (Thery et al., 2002; Bobrie et al., 2011; Fitzner et al., 2011). These findings indicate that exosomes are potential mediators of intercellular responses to inflammation and autoimmunity in CNS diseases (Kramer-Albers et al., 2007; Guescini et al., 2010; Wang et al., 2012).

Exosomes are uniquely identified by several proteins, namely, the tetraspanin proteins CD63 and CD9, lipid raft-associated proteins, including flotillin-1, the heat shock protein HSPA8, and the GTP binding protein EEF2, as well as internal, endosomal markers such as Alix and Tsg101 (Lee et al., 2012). While the contents of exosomes can also vary based on the cell of origin, exosomes routinely contain proteins, peptides, mRNA, and/or miRNA (Valadi et al., 2007). Exosomes released from one cell can fuse to another “target” cell through a non-classical active endocytotic process regulated by the lipid raft protein, caveolin-1(Svensson et al., 2013). Since exosomes are released from virtually all eukaryotic cell types, their cell-of-origin can also be identified by retained cell-phenotype specific markers (Colombo et al., 2014), which makes exosomes potentially useful as sentinels for CNS function in pathophysiology.

In this study we have focused on the emerging role of activated astrocytes as mediators and dynamic participants in a growing number of neurological diseases (Husain et al., 2011; Di Battista et al., 2015; Jany et al., 2015; Rozanski et al., 2016; Luger et al., 2017; Welch et al., 2017). To investigate the source and identity of exosomes, we have developed and validated a rigorous methodology to identify astrocyte-derived exosomes in biological samples. We also demonstrate the utility of our method by identifying astrocyte-derived exosomes in the circulating blood of mice and also report elevated detection of these astrocyte-derived exosomes in the blood of mice in an animal model of inflammatory demyelination. We hypothesize that these astrocyte-derived exosomes relay systemic signaling related to disease and therefore represents a potentially important biomarker of CNS function. The detection of astrocyte exosomes in blood also suggest that astrocytes may exert a much broader impact on homeostatic and pathogenic regulation of signaling pathways on other cells during disease.

## Materials and methods

### Animals and experimental autoimmune encephalomyelitis

All procedures involving animals were conducted with approval from the Institutional Animal Care and Use Committee at the University of Connecticut School of Medicine and in accordance with guidelines set forth by the National Research Council of the National Academies *Guide for the Care and Use of Laboratory Animals*. Mice used in this study were included wildtype C57BL/6 (strain #000664) and GFAP-Cre mice (JAX strain #024098). To induce experimental autoimmune encephalomyelitis (EAE) wild-type C57BL/6 mice (6–8 weeks old) were immunized with a 1:1 ratio of myelin oligodendrocyte glycoprotein (MOG_35−55_, AnaSpec Inc.) dissolved in deionized water and complete Freund's adjuvant (CFA, Sigma) containing 0.5 mg of Mycobacterium tuberculosis H37RA (Difco Laboratories: BD Diagnostics), as previously described (Crocker et al., 2006). The MOG-CFA emulsion was administered subcutaneously (s.c.) into the flanks of the hind-limbs (300 μg/mouse). On days 0 and 2, pertussis toxin (PTX, List Biological) was injected intraperitoneally (i.p.) (500 ng/mouse). Weights and clinical scores were recorded daily. The following grading scheme was used to score clinical signs of disease severity: 0, no clinical signs; 0.5, distal tail limpness; 1, full tail atony; 2, hindlimb paresis; 3, unilateral hindlimb paralysis; 4, bilateral hindlimb paralysis; 5, moribund. Blood was collected at peak clinical illness.

### Primary glial cultures

Cultures were generated from cerebral cortices of neonatal C57BL/6 mouse pups (P0–P3) using a neural tissue dissociation kit (Miltenyi Biotec), as previously described (Crocker et al., 2008). Cells were plated into T75 flasks. The purity of each culture was confirmed and consistent with previous reports of 90–97% GFAP+ cells (Crocker et al., 2008), as verified by immunocytochemistry (ICC) for GFAP for astrocytes (1:500, Sigma-Aldrich) and Iba-1 for microglia (1:1,000, WAKO). Cells were grown to 70–80% confluence, washed two times with PBS and incubated for 24 h in serum-free media (Dulbecco's modified eagle medium, 1% Pen-strep, Gibco) or in the presence of 10 ng/mL of IL-1β (Peprotech). Cell culture conditioned media (CCM) was collected after 24 h, spun at 3,000 × *g* for 15 min at room temperature, and stored at −80°C until use.

### Blood collection and exosome isolation

Blood was collected from CFA or EAE animals at pre-and peak clinical illness while under deep isoflurane anesthesia using a 1 ml syringe that had been flushed with 0.5 M EDTA (Fisher Scientific). Exosome isolation workflow from blood samples is shown in **Figure 3A**. Blood volumes collected ranged from 300 to 500 μl/animal. Blood was centrifuged at 2,000 × *g* for 15 min at room temperature and the upper plasma layer was drawn off. Plasma was centrifuged at 1,500 × *g* at room temperature and the supernatant drawn off. Plasma samples were then subjected to a differential ultracentrifugation protocol for exosome isolation which were confirmed by electron microscopy, western blotting, and flow cytometry. Briefly, plasma was spun at 12,000 × *g* for 30 min at 4°C. The supernatant was collected and run through a 0.22 μm spin column (Millipore) at 12,000 × *g* for 4 min at room temperature. Filtrate was collected and equal amounts were ultracentrifuged at 118,000 × *g* for 90 min at 4°C. The supernatant was removed and the exosome pellets were re-suspended in 100 μl of sterile, 0.22 μm filtered PBS(−) and stored at −80°C until use. All samples were validated by electron microscopy.

### Exoquick-TC™ precipitation

**Figure 1:**
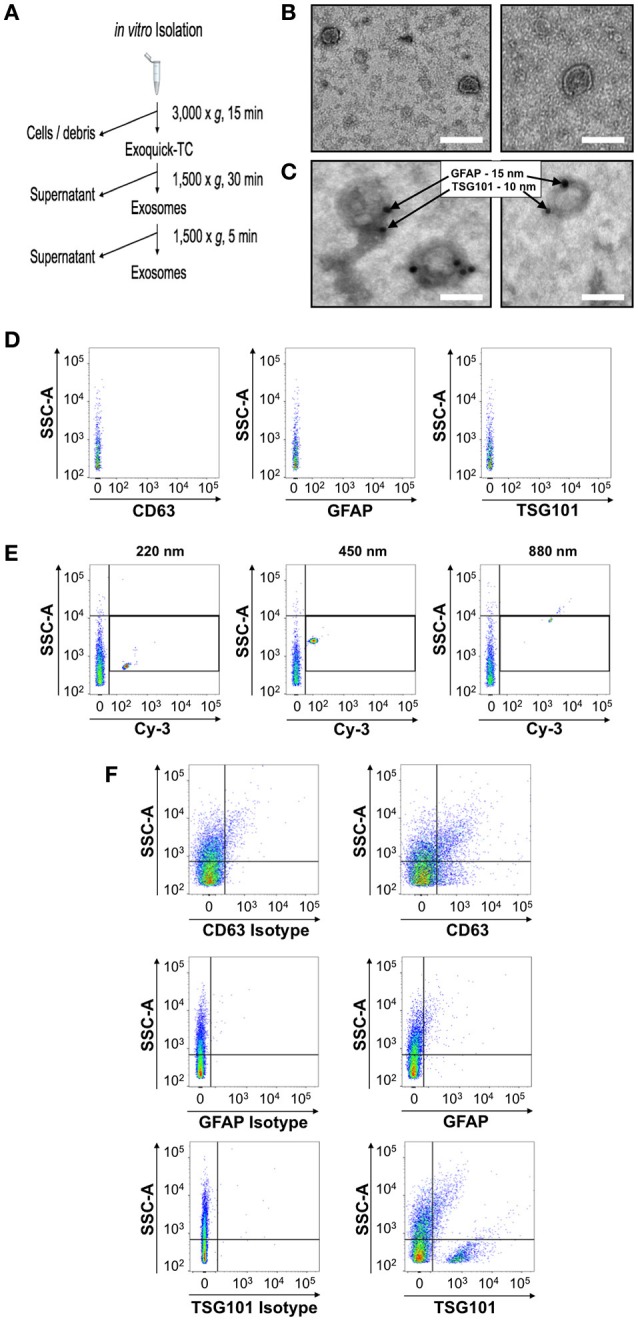
Identification and characterization of a flow cytometry methodology to identify astrocytic exosomes in media from primary astrocyte cultures. **(A)** Work-flow scheme of exosome isolation from primary culture conditioned media. **(B)** Negative stain electron micrographs of exosomes isolated from media using a commercially available kit which allows for rapid isolation and purification of exosomes **(C)** Electron micrographs of astrocyte-derived exosomes in media verified using immunogold electron microscopy against the exosome marker TSG101 (15 nm gold-particle) and astrocyte marker GFAP (10 nm gold particle). **(D)** The diluent, phosphate-buffered saline, was analyzed by the flow cytometer prior to any exosome analysis to determine the background noise of the machine prior to each exosome sample. **(E)** Yellow-fluorescent calibration beads of different known nanometer (nm) sizes were used to identify both the threshold for detection of the flow cytometer prior to the analysis of exosome samples and the relative location of that particle size class. **(F)** Exosome were stained with anti CD63, anti-GFAP and anti anti-TSG101 (right panels) or corresponding isotype controls (left panels). Serial dilutions for each antisera were performed and compared to the isotype-matched control for each, to determine the optimal dilution that would provide the best signal-to-noise ratio for flow cytometric analysis. Scale bars in **(B,C)** = 100 nm.

ExoQuick™ precipitation was carried out according the manufacturer's instructions (System Biosciences) and experimental workflow is depicted in Figure 1A. Briefly, 3 mL of conditioned media was mixed with 0.6 mL of ExoQuick-TC™ solution by inverting the tubes several times. The samples were left to incubate overnight at 4°C then centrifuged twice at 1,500 × *g* for 30 and 5 min, respectively, in order to remove the supernatant. The supernatant was discarded and the pellet was re-suspended in 100 μL of 0.22 μm-filtered PBS(−) and stored at −80°C until use.

### Electron microscopy imaging

For negative staining, 15 μl drops of exosomes (in PBS) were adsorbed onto activated copper grids with carbon coating (Electron Microscopy Sciences) for 15 min, washed by dabbing the grid onto three drops of deionized water, and stained with 1% uranyl acetate (Electron Microscopy Sciences) for 1 min. Grids were imaged under a Hitachi H-7650 transmission electron microscope. For immunogold labeling, 15 μl of isolated exosomes were adsorbed onto activated nickel grids with carbon coating for 15 min, Grids were then transferred to 100 μl drops of PBS and washed twice for 3 min each. Following the PBS wash, grids were transferred to 100 μl drops of a PBS/50 mM glycine solution and washed four times for 3 min each. Grids were then blocked for 10 min on 100 μl drops of blocking buffer (1% BSA in PBS). Grids were then incubated for 30 min at room temperature on 30 μl drops of primary antibody against TSG101 (1:10 dilution in 1% BSA; Genetex) and GFAP (1:10 dilution in 1% BSA; Millipore and Novus Biologicals). Grids were washed on 100 μl droplets of PBS three times for 5 min, blocked in 1% BSA for 5 min, and incubated with the appropriate secondary antibody conjugated with 10 nm gold particles or 15 nm gold particles (Electron Microscopy Sciences; 1:15 dilution in blocking solution) for 30 min. Grids were washed three times for 5 min in PBS followed by successive deionized water washes and counter-stained with 15 μl of 1% uranyl-acetate for 1 min. Excess uranyl-acetate was removed by gently blotting the grids and left to air dry before imaging under a transmission electron microscope. Scanning electron micrographs were performed on astrocyte in cultures that were fixed in 2.5% gluteraldehyde, post-fixed using osmium tetroxide and then dehydrated using ethanol and processed by critical point drying. Cells were then spatter-coated with gold palladium and visualized using a JOEL Scanning EM (JOEL USA Inc.).

### Western blot analysis

Exosome preparations were lysed in RIPA buffer (with protease inhibitor cocktail; Sigma) and separated by SDS-PAGE. Proteins were transferred to nitrocellulose and immunoblotted using unconjugated antisera against CD63 (GeneTex), GFAP (Sigma), or TSG101 (GeneTex) that were then visualized by HRP-conjugated secondary antisera using chemiluminescence (ECL; Amersham).

### Flow cytometry

Flow cytometric analysis on exosome surface markers was performed at the UConn Health flow cytometry core. CD63 (PE anti-mouse; 1:100; Biolegend), GFAP (Alexa Fluor 647 anti-mouse; 1:50; BD Biosciences), and TSG101 (FITC anti-mouse; 1:50; Lifespan Biosciences) were added to sterile, 0.22 μm-filtered PBS (1x, pH 7.4) and spun at 10,000 × g at 4°C for 30 min to remove protein aggregates from the staining material. Supernatants were transferred to fresh 0.2 mL PCR tubes and a 10 μl aliquot of exosome suspension was added. Tubes were vortexed before being placed in a 37°C incubator for 60 min. Stained samples were then transferred to round-bottom polystyrene tubes and analyzed using a Becton Dickinson (BD) FACS Aria-II with a 130 μm nozzle and 10 PSI. Samples were run at the lowest flow rate to ensure the most focused core stream until approximately 10,000 single events [based on a 1:1 ratio of side scatter (SSC) pulse height to pulse area] were captured. Threshold was set based upon SSC at the minimum value of 200 in order to remain unbiased for fluorescence particle detection. Noise was determined by running the diluent (0.22 μm-filtered PBS) until ~500–1,000 “noise events” were captured. This was considered the background signal for the instrument. For analysis of exosomes, a minimum of 10,000 events were captured and sorted for subsequent electron microscopy analysis. The detector voltage for Alexa Fluor 647 was set such that unlabeled EVs gave a signal that was 2x the robust standard deviation of the “noise events” to clearly distinguish the sample contents from the diluent and background signal from the instrument. This was determined by the cytometer baseline report from BD Cytometer Setup and Tracking software. Reference beads of known sizes (Spherotech, Nano Fluorescent Size Standard Kit; #NFPPS-52-4K) were also used to determine instrument performance the day's experiment as outline in section 3.1. Analysis and gating was performed using BD FACS Diva software and FlowJo V10.2.

### Statistical analyses

Experiments were performed in quadruplicate technical replicates for each of at least three biological replicates per condition. Comparisons between treatments were made using Student's *t*-test or repeated measures ANOVA with significance indicated where appropriate. Data are presented as mean ± SEM using scatter plots to demonstrate data distribution within each treatment group. The null hypothesis for all experiments was *P* < 0.05.

## Results

### Identification of astrocyte exosomes in primary cultures

To establish the identity of exosomes from astrocytes in more complex biological fluids, we first collected serum-free media from primary astrocytes in culture in order to benchmark the approach and reagents under these defined conditions. Exosomes were collected and then analyzed by transmission electron microscopy, which revealed negatively stained exosome-like double membrane vesicles with size ranges between 50 and 150 nm, with a stereotypical cup-shaped morphology (Figure 1B). The identity of these extracellular vesicles as exosomes was then confirmed by immunogold labeling with the exosome marker TSG101, in conjunction with the astrocyte marker, glial fibrillary acidic protein (GFAP) (Figure 1C). Together, these histomorphological features confirmed the collection of exosomes from the astrocyte-conditioned media.

We next sought to determine whether flow cytometry could also be adopted as a means to reliably identify exosomes in media samples conditioned by primary astrocytes in culture. Flow cytometry analysis of exosomes is presently considered a highly desirable, widely accessible technology by which to assess the presence of small vesicles, however, the standardization of this method for small particle analysis is still in development with no final consensus on the exact method to use. A significant limitation of this analysis remains the resolution of size using forward angle light scatter (FALS) to trigger a particle “event” on the flow cytometer. Due to their small size, extracellular vesicles cannot be distinguished using a traditional forward scatter signal detector or photodiode. Some customized and specialized instrumentation uses a more sensitive photomultiplier tube for FALS (Stoner et al., 2016), however not all labs have access to such instrumentation. On the other hand, side angle light scatter (SSC) and fluorescence are both detected using the more sensitive photomultiplier tube (PMT) which allows for resolution of low signals such as those produced by small vesicles with respect to the background electronic “noise” of the instrument. Taking advantage of these detectors, we set the cytometer to trigger events based on the lowest possible setting for SSC and subsequently examined the fluorescence of any particle that registered above the noise of the instrument for the given fluorescence detector. Noise was determined by acquiring a sample of the media used to dilute the vesicles (Figure 1D). The instrument performance for this detection method was determined using a set of yellow-fluorescent nano-calibration beads on each experimental day to ensure detection of a 200 nm particle (Figure 1E). Although polystyrene bead particles do not have the same refractive index as lipid vesicles for the generation of SSC, this test ensured the instrument was at least capable of detecting small particles above noise. Another caveat to the use of flow cytometry for small particle detection is the possibility of larger aggregates of particles (“swarm detection”), where several vesicles were detected simultaneously (van der Pol et al., 2014). To reduce this possibility, all exosome samples were run both undiluted and diluted to ensure the fluorescence intensity of the signal remained constant. In addition to the dilution of the sample, each exosome sample was run simultaneously with an unstained sample, a sample allowed to react with an isotype control Ab, and a sample which only contained antibody with dilution media. This strategy then enabled us to discern the labeled exosome signal from aberrant signal from unbound antisera and also non-specific binding of Ab to exosomes (Figure 1F). Using this step-wise approach we identified exosomes bound to Abs specific for the astrocyte marker GFAP as well as canonical exosome markers TSG101 and the tetraspanin protein, CD63 (Figure 1F).

### Increased astrocyte exosome release in response to IL-1β

**Figure 2:**
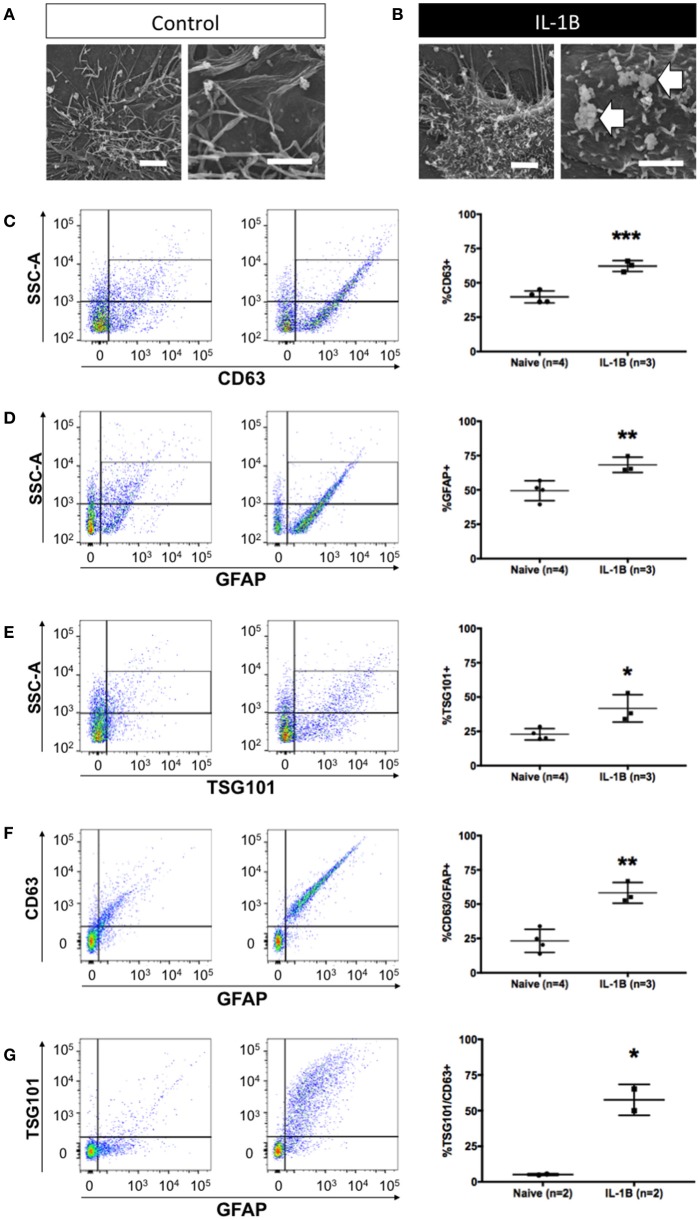
Quantitative increase in detection of astrocytic exosomes media from primary astrocyte cultures following treatment with IL-1β. Scanning electron micrographs of astrocytes under control conditions **(A)** and after 1 h after IL-1B-stimulation **(B)**. Magnifications in A&B are: 6,500x, left panel, and 10,000x, right panel). Note (arrows) the membrane perturbations resulting from cytokine stimulation that have been associated with process of extracellular vesicle release **(B)**. **(C–G)** Analysis of astrocyte-derived exosomes from primary glial cultures using flow cytometry using antibodies against CD63 **(C)**, GFAP **(D)**, and Tsg101 **(E)** show elevated detection of events (exosomes) following IL-1B treatment. Additional analyses of GFAP+/CD63+ **(F)** and GFAP+/Tsg101+ **(G)** labeling confirmed an increased relative abundance of astrocyte-derived (GFAP+) exosomes (CD63+ or Tsg101+). Control (vehicle) treated cultures (“Naive”) had an identifiable amount of exosomes in conditioned media but the proportion of exosomes detected was markedly increased in response to IL-1β treatment. Significance is indicated where: ^*^P < 0.05; ^**^P < 0.01, ^***^P < 0.001 *t*-test. Scale bars in **(A,B)** = 1 μm.

We next tested whether our methodology could be used to identify changes in exosome release. Previous work has shown that IL-1β is a potent inducer of exosome release. We began by examining astrocytes using scanning electron microscopy to determine if membrane perturbations/projections, associated with extracellular vesicle release in other cell types could be observed in astrocytes. In untreated astrocytes (Figure 2A), the plasma membrane was observed to have many long, sinewy projections. In contrast, shortly after treatment with IL-1β, the cell surface of astrocytes was noticeably different with numerous bulbous protrusions which resemble microblebs (Figure 2B), which reflect intracellular budding events consistent with the process of exosome release from exocytosis of MVBs (Gyorgy et al., 2011). This subcellular structural response was then associated with a measurable increase in the abundance of exosomes into the cell culture media (Figures 2C–G). To test if our flow cytometry approach could be used to identify exosomes release from astrocytes activated by IL-1β we assayed the proportion of exosomes released into the conditioned media collected 24 h after treatment. IL-1β treatment significantly increased the abundance of CD63+ particles smaller than 220 nm (exosomes) (Figure 2C, *P* < 0.001). The proportion of exosomes released by astrocytes was also increased (Figure 2D, *P* < 0.01), and we confirmed the exosomal origin of these extracellular vesicles using a second marker Tsg101, which also identified a significant increase in response to IL-1β treatment (Figure 2E, *P* < 0.05). We further validated the identity of increased exosomes as having been derived from astrocytes by determining the relative increase in the proportion of GFAP+/CD63+ (Figure 2F, *P* < 0.01) and GFAP+/TSG101+ exosomes (Figure 2G, *P* < 0.05). Thus, we confirmed the utility of all three markers of astrocyte-derived exosomes (GFAP, CD63 and TSG101) and determined that these markers exhibited remarkable concordance between each marker in terms of its magnitude of increase in response to IL-1β treatment. Once again, for each exosome isolate tested, we verified by TEM the appropriate size class of extracellular vesicles collected from each sample (data not shown).

### Identification of astrocyte exosomes in peripheral blood samples

The objective of developing a validated approach to identify exosomes from primary astrocytes conditioned media was to be able to apply this approach to more complex biological fluids. Interestingly, the astrocyte marker protein GFAP has been reported to be found in blood from patients suffering from a number of neurodegenerative diseases including Alzheimer's, ALS, and multiple sclerosis (Malmestrom et al., 2003; Mayer et al., 2013). Therefore, we hypothesized that GFAP identified in blood samples may indicate the presence of astrocyte-derived exosomes. This would suggest that astrocyte-derived exosomes are present in peripheral circulation.

**Figure 3:**
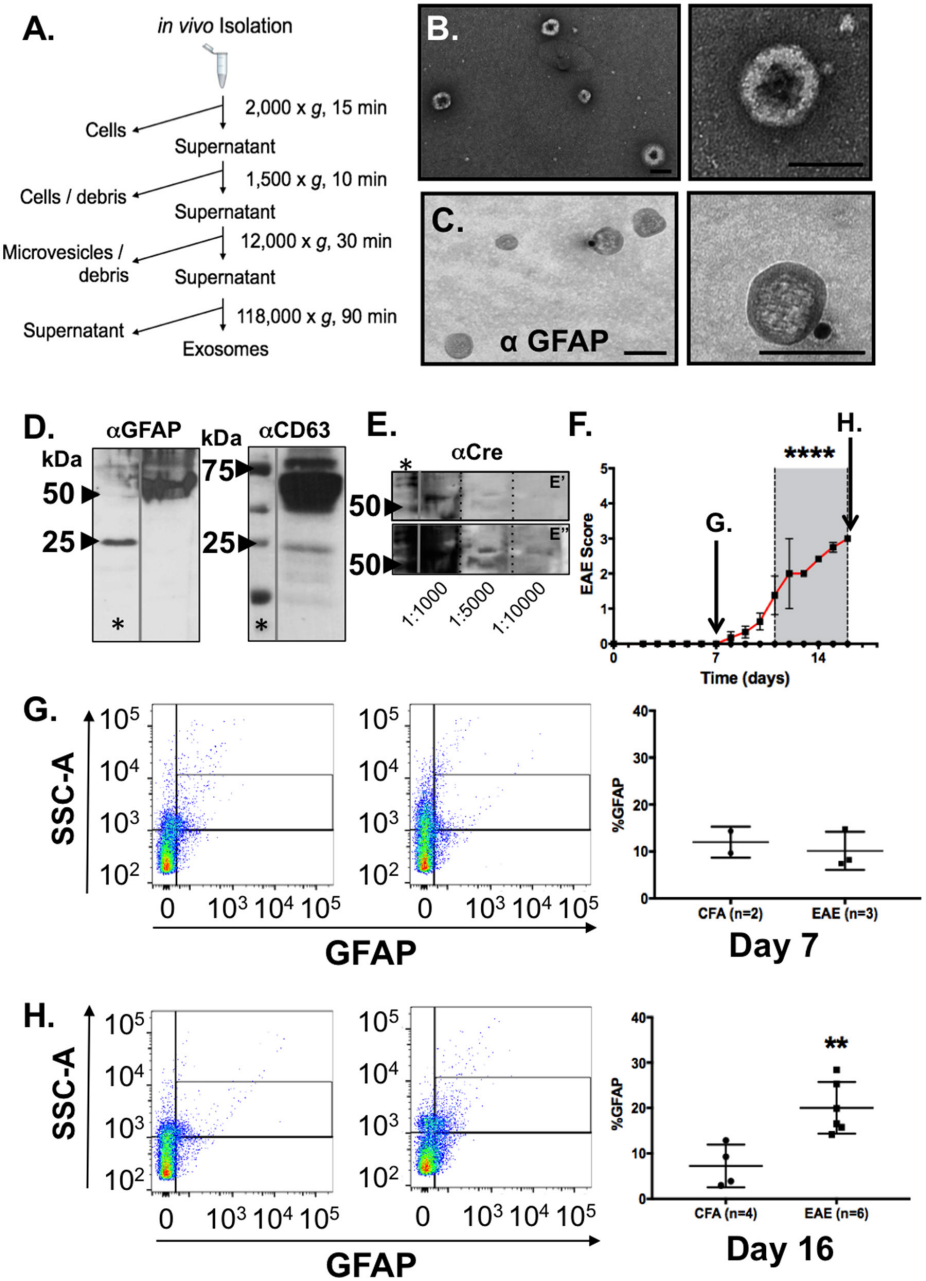
Identification and characterization of astrocyte-derived exosomes in blood plasma from mice and increased detection of GFAP+ exosomes in blood from mice during EAE. **(A)** Work flow scheme for exosome isolation from blood plasma. **(B)** Electron micrographs of extracellular vesicles isolated from peripheral blood plasma revealed prototypic “cup-shaped morphology” of the appropriate size class for exosomes. **(C)** Immunogold electron microscopy using anti-GFAP and gold particle conjugated secondary antisera (15 nm) identified exosomes from blood plasma that were astrocyte-derived. Scale bars: size bar, **(A)** = 100 nm; **(B)** = 100 nm. **(D)** Western blotting analysis of the EM exosome preparations from blood plasma analyzed in **(A,B)** confirmed detection of exosomal (CD63) and astrocytic markers (GFAP) in the exosome pellet. **(E)** Validation of astrocytic origins using expression of CRE recombinase protein in blood plasma as measured by western blotting from exosome preparation from GFAP-CRE transgenic mice. Samples were diluted (as indicated) to demonstrate that antibody reactivity was serially diminished which supports the specificity of the antibody binding. **(E')** represents a shorter exposure of the blot, while **(E”)** represents a longer exposure time which were needed to visualize CRE-reactive bands in the more diluted samples, respectively. The CRE-reactive bands were of the same molecular weight as observed in the most concentrated sample. **(F)** Clinical EAE scores of myelin oligodendrocyte glycoprotein (MOG) immunized C57BL/6 mice (*n* = 6) and control (CFA inoculated; *n* = 4). Mice were euthanized one week following immunization or time point of peak clinical illness (Day 16). Euthanization of CFA control animals were time-matched for either day 7 (arrow) or day 16 (arrow), respectively, for blood collection and blood plasma exosome isolation. **(G)** Flow cytometry analysis of blood plasma exosomes from EAE and control (CFA) mice at a pre-clinical disease timepoint (Day 7), and **(H)** at the time of peak clinical illness (Day 16). GFAP+ exosomes in blood of control mice did not differ in preclinical disease (Day 7) MOG immunized mice, whereas a notable increase in the detection of astrocyte-derived exosomes in blood plasma was observed in mice at the time of peak clinical illness in EAE. Significance is indicated where: **(F)** ^****^P < 0.0001, 2-way ANOVA; **(H)** ^**^P = 0.0031; *t*-test.

To test this possibility, we collected blood samples from naïve C57BL/6 mice and isolated exosomes from blood plasma using a differential centrifugation protocol (Witwer et al., 2013). Exosomes isolated by this method (Figure 3A) were analyzed by TEM to determine if the vesicles present had all the characteristics of exosomes we observed in our conditioned media samples. Negative staining confirmed a distinctive cup-shaped morphology of the double membrane vesicles and size class consistent with exosomes, ranging from 50 to 150 nm size range, in these blood plasma samples (Figure 3B). Immunogold labeling of exosomes with the astrocyte marker GFAP identified astrocyte-derived exosomes in these samples from peripheral blood (Figure 3C).

To address whether these GFAP-positive exosomes in blood were indeed derived from astrocytes in the CNS and not from other peripheral tissues that have been suggested to be immunoreactive for GFAP (Malmestrom et al., 2003), we collected blood samples from CRE recombinase mice in where the transgene was under the control of the mouse GFAP promoter [B6.Cg-Tg(Gfap-cre)73.12Mvs/J] (Garcia et al., 2004). CRE mRNA expression in these mice is limited to the brain and spinal cord whereas no CRE expression is detected in peripheral tissues (data not shown). Blood plasma samples were collected and the exosome pellet subjected to western blot analysis for the exosome marker CD63, as well as astrocyte specific markers GFAP and the transgene product, CRE protein. This western blot analysis confirmed both expression of exosome marker, CD63 (Figure 3D), as well as the astrocyte markers GFAP (Figure 3D) and the gene product of the transgene, CRE (Figure 3E). Together, these findings confirmed that GFAP+ exosomes present in peripheral blood circulation of naÏve adult mice were of astrocytic origin.

### Elevated astrocyte-derived exosomes in blood from clinically affected EAE mice

We next examined whether the amount of astrocyte exosomes in peripheral blood changed during course of clinical disease in a model of a neurological disease. To test this, we used the same strain of C57BL/6 mice for which we derived our primary glial cultures, and immunized adult mice to induce EAE using myelin oligodendrocyte glycoprotein (MOG) peptide as described above. This model of inflammatory demyelination induces a robust, reproducible, and predictable CNS demyelination that is used to model the immune-mediated pathology of multiple sclerosis. In this set of experiments, mice immunized with MOG_35−55_ peptide, or given an equal volume of CFA, were monitored daily for signs of clinical illness and at the peak clinical disability (Figure 3F), Blood was collected from mice either 1 week following immunization with MOG, or at the peak of clinical disability (Day 16) post immunization (Figure 3F). Blood samples from CFA-treatment groups were time-matched to either 7 or 16 day time points (Figures 3G,H) Exosome samples were then analyzed using flow cytometry to determine the abundance of GFAP+ exosomes in the EAE group when compared to CFA controls. Flow cytometry identified increased a low abundance of GFAP+ exosomes in all samples and the relative abundance in CFA-treated animals did not differ from MOG-immunized animals 7 days post injection (Figure 3G; *n* = 4–6/group). In contrast, analysis of GFAP+ exosomes from blood of EAE mice taken during peak clinical disability (Day 16) revealed a significant increase in the relative abundance of GFAP+_exosomes in EAE mice when compared with time-matched CFA-only treated controls (Figure 3H). Taken together, these data indicate that neuroinflammation associated with EAE was associated with increased presence of astrocyte-derived exosomes in the blood.

## Discussion

Accumulating evidence indicates that astrocyte dysfunction contributes to the pathogenesis of several prevalent neurodegenerative diseases (Maragakis and Rothstein, 2006; Williams et al., 2007; Halliday and Stevens, 2011; Verkhratsky et al., 2012), including Alzheimer's disease (Adelman et al., 2013), amyotrophic lateral sclerosis (ALS) (Ilieva et al., 2009), Parkinson's disease (Teismann et al., 2003; Gu et al., 2010), Huntington's disease (Bradford et al., 2009, 2010; Faideau et al., 2010), Alexander's disease (Verkhratsky et al., 2012), and multiple sclerosis (Williams et al., 2007; Moore et al., 2011b; Yu et al., 2013). Alterations in astrocyte function are also reflected in their exosome function. For instance, motor neuron death in ALS is non-cell autonomous and results from toxic secreted factors from astrocytes (Nagai et al., 2007; Diaz-Amarilla et al., 2011; Haidet-Phillips et al., 2011; Papadeas et al., 2011). Exosomes collected from SOD1 mutant astrocytes have been found to contain the mutant SOD1 protein, that is transferred into cultured motor neurons when the exosomes fuse (Basso et al., 2013). Exosome-mediated transfer of apoptotic proteins also provides a potential mechanism for glial cell death Alzheimer's disease (Wang et al., 2012). The contents of astrocyte exosomes are unknown, but given the important physiological roles for astrocytes in neural development (McCall et al., 1996; Pacey and Doering, 2007; Hochstim et al., 2008), myelination (Watkins et al., 2008; Moore et al., 2011b), synaptic transmission (Christopherson et al., 2005; Henneberger and Rusakov, 2010), neurovascular coupling (Voskuhl et al., 2009; Petzold and Murthy, 2011), regulation of CNS infections (Zheng et al., 2001; Aubagnac et al., 2002; Wilson and Hunter, 2004; Ramesh et al., 2009), and inflammation (Liedtke et al., 1998; Crocker et al., 2006; Carpentier et al., 2008; Toft-Hansen et al., 2011). The context of astrocyte activation in disease has lead to proposed phenotype classification with “A1” pro-inflammatory and “A2” anti-inflammatory states (Nash et al., 2011; Tarassishin et al., 2011; Chaboub and Deneen, 2012; Rusnakova et al., 2013; Sosunov et al., 2013; Liddelow et al., 2017). Our *in vitro* experiments using primary astrocytes in culture indicate that the abundance of exosomes can be increased in response to IL-1β, which is may be indicative of an “A1” state, whereas our *in vivo* findings may reflect activation but perhaps a mix of A1 and A2 states. Nevertheless, our approach to study exosomes from astrocytes may be applied to qualify the exosomal components related to these different activation states. Our findings demonstrate that astrocytes respond to pro-inflammatory conditions by increasing the release of exosomes. Our new validated protocol for detecting astrocyte-derived exosomes in conditioned media has also enabled us to assay astrocyte-derived exosomes in more complex biological samples (i.e., blood plasma).

In this study, we focused on the potential utility of identifying astrocyte exosomes as they may relate to study of their function in primary culture and analysis of these extracellular vesicles in blood, by applying our methods to an animal model of multiple sclerosis (MS). As mentioned, MS is a progressive, demyelinating neurodegenerative disease characterized by development of autoreactive T cells, CNS demyelination, and chronic astrogliosis. A number of studies suggest that reactive astrocytes contribute heavily to the disease process of demyelination (Moore et al., 2011a; Correale and Farez, 2015; Li et al., 2016). The interplay between the central nervous system and the immune system in the development and progression of MS, and in particular the potential role of astrocytes as regulators of T cell activities in the CNS raises intriguing questions about the many ways in which astrocyte dysfunction may contribute to pathology in this disease (Huseby et al., 2015; Rothhammer and Quintana, 2015). Importantly, how these systems counter-regulate during the course of disease is a matter currently under investigation.

Our findings are the first to identify a potentially new and precise process through which the CNS and immune systems may interact: the release of activated, A1-type astrocyte (GFAP+) exosomes into the blood may indicate a previously unrecognized, wide-ranging influence of CNS astrocytes on systemic physiology in disease. Our findings also identified GFAP positive exosomes in the peripheral circulation of mice and increased levels of circulating exosomes in the mouse model of multiple sclerosis, EAE. This suggests that astrocytes may exert a much broader impact on homeostatic and pathogenic regulation of signaling pathways on other cells during disease. While we have not determined whether these changes reflect an alteration in the physiological function of exosomes released constitutively versus in response to inflammation, we would hypothesize, based on the known function of astrocytes in disease models, that the content and function of exosomes would mirror the (patho)physiology of the CNS. For this reason we would propose that a potential development from these findings would be the identification of specific content markers from within these exosomes that could be used as a biomarker to monitor the state of the CNS in health or disease.

Experimental evidence for a contributing role of exosomes to the progression of multiple sclerosis is currently limited, yet studies identifying the presence of extracellular vesicles in numerous body fluids including cerebrospinal fluid (Scolding et al., 1989), blood (Minagar et al., 2001), and urine (Giovannelli et al., 2015). The findings suggest that monitoring of CNS-derived extracellular vesicles, including exosomes, may offer utility for monitoring disease progression and/or responsiveness of MS patients to therapies. For instance, Minager et al. observed an increase in endothelial derived microparticles in the plasma of MS patients during a relapse that showed a subsequent reduction to control levels during remission (Minagar et al., 2001). Exosomes have also been investigated for use as a potential therapeutic to stimulate remyelination in MS patients as evidenced by the identification of micro-RNA (miRNA) species within the cargo of exosomes that can improve remyelination. This evidence is based off the finding that exosomes isolated from interferon-γ stimulated rat bone marrow derived dendritic cells containing miRNA-219, which is capable of increasing baseline myelination, reducing oxidative stress, and improving remyelination in rats following a lysolecithin demyelinating injury (Pusic et al., 2014). These exosomes could also be administered intranasally to rats to improve myelination. Beyond a potential therapeutic use of exosomes in MS, there is evidence for their use a diagnostic marker in patients receiving treatments. miRNA species have been detected in the blood and urine of MS patients undergoing natalizumab therapy (Giovannelli et al., 2015).

The aforementioned findings provide additional future avenues of investigation for the potential of astrocyte-derived exosomes in peripheral blood circulation in a wide variety of neurological conditions in which changes in astrocytes have been implicated. Currently, exosomes are often viewed as passive players: a reflection of the development and progression of numerous neurodegenerative diseases. Yet, recent studies provide compelling evidence for an active, participatory role for exosomes, including astrocyte-derived exosomes, in chronic neurodegenerative diseases. Astrocyte-derived exosomes have already been implicated in contributing to the progression and pathogenesis of amytrophic lateral sclerosis (ALS) (Maragakis and Rothstein, 2006; Sofroniew and Vinters, 2010), Alzheimer's (Maragakis and Rothstein, 2006), and Parkinson's (Maragakis and Rothstein, 2006). In each of these neurodegenerative diseases, astrocyte exosomes were shown to carry the mutant version of superoxide dismutase-1 enzyme (Basso et al., 2013), amyloid precursor protein (Rajendran et al., 2006), and alpha-synuclein (Shi et al., 2014), respectively. However, there has been no definitive evidence for a role of astrocyte exosomes in contributing to the pathogenesis of multiple sclerosis. This is perhaps due to evidence for an active and dual-role of astrocytes in MS pathogenesis. It is known that astrocytes play diverse roles in CNS development and disease, but in MS, specifically, astrocytes garner attention for their potential to enhance immune responses through expression of major histocompatibility complex (MHC) class I and class II molecules (Zeinstra et al., 2003; Höftberger et al., 2004) and activation of CD8^+^ and CD4^+^ T cells (Sedgwick et al., 1991; Nikcevich et al., 1997; Tan et al., 1998). Conversely, astrocytes may also elicit neuroprotective functions: release of anti-inflammatory molecules (i.e., TGF-β), supporting oligodendrocyte regeneration, and facilitating repair of the blood-brain-barrier (Sofroniew and Vinters, 2010). Understanding the precise function of astrocytes may be revealed by the exosomes they shed in response to CNS injury. Future experiments should consider the cargo of astrocyte exosomes and their potential functional impact. One of our hypotheses is that the effect of astrocyte exosomes is a function of time. It could be that early on in the disease process astrocyte exosomes have a negative role in modulating the immune response, which results in the demyelinated lesions and relapses commonly observed with MS. As the immune response lessens and patients enter remission, astrocyte exosomes could have a positive role in modulating the recovery of the lesions vis-à-vis remyelination. Future studies will be required to better understand this interesting dual-role of astrocyte exosomes in the disease process.

Presently, the cause of multiple sclerosis is not known. Our finding of elevated numbers of astrocyte-exosomes in peripheral circulation during homeostasis and during disease creates a potentially interesting avenue of study into better understanding the disease process. These findings provide a new perspective by which we may better understand this disease and potentially develop new approaches to monitor the course of and understand the pathophysiology of diseases like multiple sclerosis.

## Author contributions

CW performed experiments, analyzed the data and wrote the manuscript. AM assisted in the experimental design of the study, analyzed data, and edited the manuscript. EJ performed experiments and provided expertise in developing the flow cytometry protocol used in this report, analyzed the data, and participating in writing the manuscript. AN performed experiments, assisted in data analysis and edited the manuscript. AV and SC conceived the study and designed the experiments. AV analyzed data and edited the manuscript. SC assisted with experiments, analyzed data and wrote the manuscript with CW.

### Conflict of interest statement

The authors declare that the research was conducted in the absence of any commercial or financial relationships that could be construed as a potential conflict of interest.
